# Synergistic anti-icing and snow-melting performance of two-component road markings enabled by PCMs and slow-release salts

**DOI:** 10.1371/journal.pone.0341054

**Published:** 2026-02-02

**Authors:** Wei Zhang, Kaibo Yang, Renshan Chen, Chong Wang, Xusheng Wei

**Affiliations:** 1 School of Traffic Civil Engineering, Shandong Jiaotong University, Jinan, Shandong, China; 2 School of Transportation and Logistics Engineering, Shandong Jiaotong University, Jinan, Shandong, China; Shandong University of Technology, CHINA

## Abstract

Conventional road marking coatings suffer significant performance deterioration under winter conditions, including frost coverage, reduced retroreflectivity, and low-temperature embrittlement. This study presents a functional two-component road marking coating incorporating a composite anti-icing additive composed of a temperature-regulating phase-change material (TH-ME5) and a salt-based slow-release agent (T-SEN). The influence of additive content and the TH-ME5/T-SEN ratio on coating properties, road performance, and ice/snow mitigation was systematically evaluated. Results show that a total additive content of ≤20 wt.% maintains compliance with standard requirements for adhesion, flexibility, wear resistance, drying time, retroreflectivity, hiding power, alkali resistance, and UV aging. Ice adhesion tests reveal a two-stage anti-icing mechanism: TH-ME5 provides latent heat buffering during early freezing, while T-SEN governs long-term deicing. The optimal formulation—20 wt.% additive with a TH-ME5:T-SEN ratio of 1:3—achieved the lowest relative ice adhesion. Snow-melting simulations further demonstrate the coating’s ability to delay ice formation and reduce surface snow accumulation. This PCM–salt synergistic approach provides a feasible and scalable strategy for durable, self-deicing pavement markings in cold regions.

## Introduction

**Fig 1:**
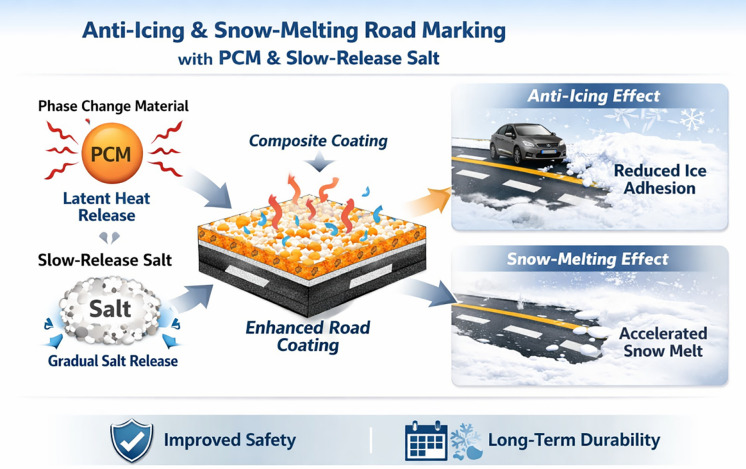
Graphical abstract. During the initial low-temperature stage, TH-ME5 undergoes phase transition and absorbs or releases latent heat, thereby moderating surface temperature changes and delaying ice formation. With prolonged exposure and further temperature reduction, T-SEN gradually releases salt ions, which lower the freezing point of interfacial water and reduce ice–coating adhesion. The combined action of TH-ME5 and T-SEN provides a sustained anti-icing and snow-melting effect without compromising the mechanical and road performance of the marking coating.

As a vital component of the national economy and a core element of modern transportation, the highway sector has experienced steady development alongside rapid economic growth and rising living standards. Road marking coatings are rapidly evolving toward diversification and multifunctionality, while the construction of highways and bridges also presents new opportunities for the industry [1]. As an essential part of traffic safety facilities, road markings provide visual information that guides traffic flow and ensures the safety of both drivers and pedestrians [2] (Fig 1).

Many researchers [3] have focused on areas such as intersections, unlit road sections, highway entrances and exits, ramps, and parking lots – locations characterized by high traffic volume, extensive marking coverage, and complex signaling. They have developed self-luminous road markings capable of adapting to comprehensive transportation scenarios, thereby enhancing integrated service functions in specific traffic contexts [4]. Examples include high-visibility markings for rainy nights, which address poor visibility caused by specular reflection under wet conditions by offering improved reflectivity and skid resistance [5]. Additionally, traffic management authorities can incorporate colored pigments into road markings to designate dedicated lanes and specific signs in various colors. When applied at tunnel portals, ramps, and sections of lower-grade roads, these markings not provide anti-slip properties but also serve as effective traffic warnings [6].

Domestic and international scholars have conducted extensive research on the road performance and multifunctionality of two-component road marking coatings. Regarding two-component road markings, Gu [7] studied and concluded that such coatings can effectively address issues such as non-environmental friendliness and lack of wear resistance during road marking construction, thereby significantly improving the quality and standards of road marking practices in China. Zhang [8], through exploratory application and follow-up testing in specific projects, demonstrated that two-component coatings outperform thermoplastic markings in terms of skid resistance, wear resistance, visibility, and environmental friendliness. Qian [9] reviewed the road performance of two-component road markings, analyzed their material systems and curing mechanisms, and discussed the impact of factors such as temperature, rainwater erosion, UV radiation, and vehicle wear on their durability. Guan et al. [10] pointed out that MMA-based two-component road marking materials differ from common solid thermoplastic coatings in that they require no heating or melting and produce no carbon emissions; instead, they form films through chemical cross-linking reactions without releasing solvents, thereby reducing emissions of organic compounds. Liu [11] developed a specialized epoxy two-component marking coating for cement pavements, with test results indicating excellent performance in practical road applications. Duan [12] compared and analyzed experimental data along with engineering application cases, suggesting that thermoplastic reflective markings are more suitable for secondary and tertiary roads in rain-rich southern regions.

In China, three-quarters of the country experiences snow cover in winter, and snow cover in some areas can last for 3–4 months. This snow and freezing phenomenon usually seriously affects the traffic warning effect of the markings, increases the driver’s driving risk and the risk of identifying the direction, and is prone to traffic accidents.

To address this issue, researchers in recent years have begun to explore and develop road marking coatings with anti-icing and snow-melting functionalities. Phase-change temperature-regulating materials and salt slow-release materials have become research hotspots due to their unique properties. Phase-change materials can absorb or release heat during temperature changes, thereby regulating the ambient temperature. Li [13] used ANF as an emulsion stabilizer and shell material, with a phase-change material as the core, to construct phase-change microcapsules. Using these as a template, a three-dimensional porous phase-change material was prepared. Experimental results showed that after 50 heating-cooling cycles, the retention rates of endothermic and exothermic enthalpy reached 97.3% and 99.0%, respectively. The porous phase-change material demonstrated excellent temperature-regulating performance and cyclic temperature control stability, making it well-suited for various application scenarios. Jiang et al. [14] adopted a self-assembly process to prepare micron-sized microcapsules using paraffin as the core and CaCO₃ as the shell. The process is relatively simple, and the performance of the microcapsules is significantly affected by the pH of the emulsion. The results indicated that the microcapsules perform best at pH 7, with a corresponding particle size of less than 50μm, meeting road application requirements. They also exhibited high encapsulation efficiency, thermal conductivity, thermal stability, and good mechanical properties.

Although two-component road marking coatings have been widely studied for durability and visibility, their long-term performance under low-temperature, snow, and ice conditions remains inadequately understood. Existing anti-icing approaches for road markings are mainly based on single mechanisms, such as salt-based additives or phase-change materials, which suffer from rapid depletion or limited effective duration, respectively. Moreover, the synergistic anti-icing behavior of phase-change materials and slow-release salts within polymer-based two-component marking systems has not been systematically investigated, particularly in terms of ice adhesion evolution under freeze – thaw cycling. Consequently, clear formulation guidelines that balance anti-icing effectiveness with essential road performance and durability requirements are still lacking.

## Materials and methods

### Materials

This two-component road marking coating system consists of Component A, which is composed of film-forming resins, pigments, fillers, and additives [15], and Component B, which is the curing agent. Polymethyl methacrylate (PMMA) resin is used as the main film-forming material. Titanium dioxide, silica powder, heavy calcium carbonate, and quartz sand are used as secondary fillers and pigments. Glass beads (Type I) are used to achieve reflectivity.

The phase-change material (PCM) was TH-ME5 microencapsulated phase-change material, and the slow-release anti-icing agent was T-SEN temperature-sensitive salt-based material. Both additives were supplied as white powders at room temperature. The other main experimental materials and manufacturers are listed in Table 1.

**Table 1:** Main experimental materials. List of raw materials, models, and manufacturers for the two-component road marking coating system.

Material	Model	Manufacturer
PMMA Resin	Polymethyl Methacrylate	Shandong Hongxu Chemical Co., Ltd.
Titanium Dioxide	Rutile Type	Shandong Lubei Chemical Co., Ltd.
Heavy Calcium Carbonate	R800	Zibo Shengming Calcium Industry Co., Ltd.
Quartz Sand	Standard Grade	Zibo Shengming Calcium Industry Co., Ltd.
Glass Beads	HT-01	Jiangsu Mingji New Materials Co., Ltd.
Dispersing Agent	Polyvinyl Alcohol Type	Jiangsu Nanrui New Materials Technology Co., Ltd.
Anti-Settling Agent	F40	Jiangsu Nanrui New Materials Technology Co., Ltd.
Bentonite	BG-8	Shandong Yonglian New Materials Technology Co., Ltd.
Curing Agent	BPO	Ruiyuan New Materials Co., Ltd.
Phase Change Material	TH-ME5	Hubei Sermer New Energy Technology Co., Ltd.
Slow-Release Salt Material	T-SEN	Jiangsu Lvxin New Material Technology Co., Ltd.

Heavy calcium powder is the predominant filler in two-component road marking coatings. Owing to the similar physical and chemical characteristics of heavy calcium powder and the composite anti-icing material—particularly comparable particle size (mesh) and color—the composite anti-icing material was incorporated as a partial replacement for heavy calcium powder, serving as a secondary film-forming component. This substitution not only enables effective heat absorption and release under low-temperature conditions but also maintains the compressive strength and whiteness required for road marking performance. Furthermore, the anti-icing material reduces the freezing point of snowmelt, thereby delaying ice formation and decreasing the ice–coating interfacial bonding strength. These effects collectively enhance the anti-skid performance of road markings and significantly improve driving safety during early-stage snowfall in winter conditions. The types of two-component road marking specimens prepared in this study are summarized in Table 2.

**Table 2:** Types of anti-icing and snow melting two-component road marking specimens. Replacement ratios of heavy calcium powder and addition ratios of PCMs and slow-release salt materials.

Number	Heavy Calcium Powder Replacement Ratio (%)	Phase Change Material Addition Ratio (%)	Slow-Release Salt Material Addition Ratio (%)
1	0	0	0
2	10	25	75
3		50	50
4		75	25
5	20	25	75
6		50	50
7		75	25
8	30	25	75
9		50	50
10		75	25
11	40	25	75
12		50	50
13		75	25
14	50	25	75
15		50	50
16		75	25

### Methods

#### Sample preparation.

The composite anti-icing additive was prepared by physical blending according to the proportions listed in Table 2, comprising TH-ME5 phase change materials (PCMs) and T-SEN slow-release salt. Component A was homogenized using a mechanical stirrer at 500 rpm for 10 min, during which the PMMA resin, pigments, fillers, and composite anti-icing additive were thoroughly mixed to ensure uniform dispersion. Subsequently, Component B (curing agent) was introduced and mixed for an additional 3 min.

**Fig 2:**
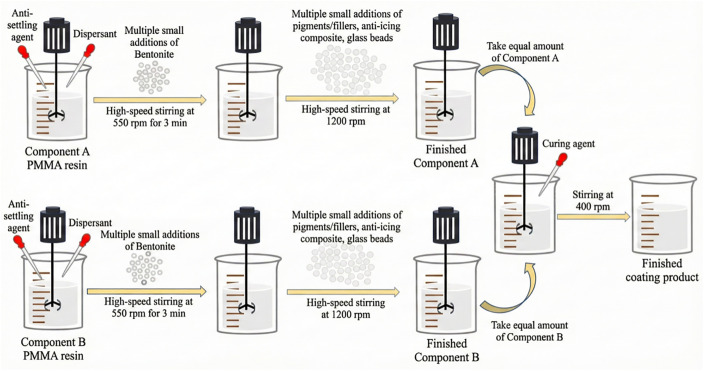
Adhesion test chart. The figure illustrates the preparation procedure of the anti-icing and snow-melting two-component road marking coating.The phase-change material (TH-ME5) and the slow-release salt agent (T-SEN) are first physically blended at the designed ratio to form a composite anti-icing additive. The additive is then mixed with PMMA resin, pigments, and fillers to form Component A and homogenized by mechanical stirring. Subsequently, Component B (curing agent) is added and mixed to initiate curing. The resulting coating is applied onto the substrate and cured under standard conditions to obtain specimens for subsequent performance and anti-icing/snow-melting tests.

The coating was applied to pre-cleaned glass substrates, metal plates, or fiber cement boards using a film applicator (draw-down applicator). All specimens were cured for 24 h under standard laboratory conditions of (23 ± 2)°C and (50 ± 5)% relative humidity prior to testing. For comparison, an unmodified two-component road marking coating without the anti-icing additive was prepared under identical conditions and used as the control. The schematic preparation procedure is illustrated in Fig 2.

#### Coating performance test of marking paint.

**Adhesion strength**

**Fig 3:**
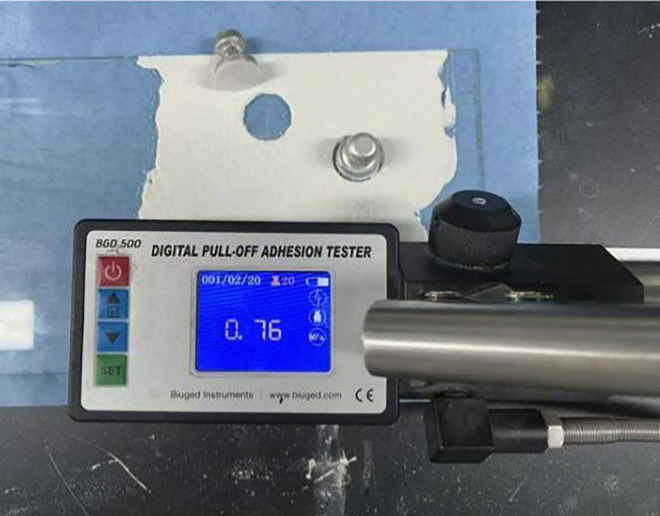
Adhesion test chart.

Although the conventional cross-cut (circle-drawing) test specified in GB/T 1720–2020 [16] provides a qualitative assessment of coating adhesion, its limited resolution makes it insufficient for distinguishing subtle adhesion differences among the various coating formulations. Therefore, adhesion strength was quantitatively evaluated using a pull-off test. After curing, pull-off dollies were bonded to the coating surface with a high-strength adhesive and allowed to cure for an additional 24h. The maximum pull-off force at failure was recorded, and the adhesion strength was calculated based on the measured failure load and the bonded area. The tests were conducted using a BGD 500 digital pull-off adhesion tester (Dongguan Langrui Instrument Co., Ltd., China), as illustrated in Fig 3.

**Flexibility**

**Fig 4:**
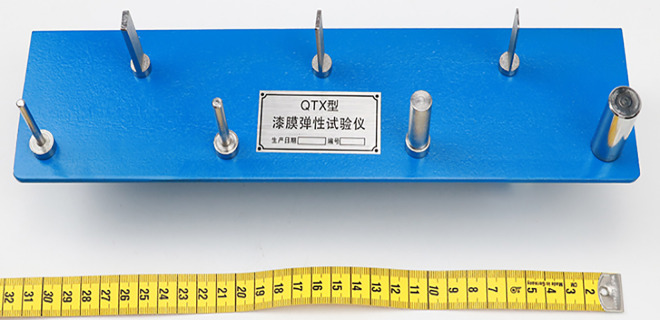
Paint film flexibility and elasticity bending tester.

The flexibility of the coating film was evaluated in accordance with GB/T 1731–2020, Determination of Flexibility of Paint Film and Putty Film [17]. Thin metal test panels with dimensions of 200 mm × 60 mm were prepared. The marking paint was applied to the metal substrates without the addition of glass beads, followed by the same curing procedure described previously. After curing, each coated panel was manually bent over a mandrel with a specified diameter using a film flexibility tester. The panel was bent at a steady rate within 2-3s by symmetrically placing both thumbs along the centerline of the mandrel. The coating was then examined for the presence of reticulation, cracking, or peeling. The flexibility tests were performed using a QTX film flexibility tester (Chengdu Zhicheng Tongchuang Electromechanical Equipment Co., Ltd., China), as shown in Fig 4.

**Non-stick tire time**

The non-stick tire time provides a direct indication of the drying rate of road marking coatings, reflecting the kinetics of solvent evaporation and the curing of film-forming components. For solvent-based marking coatings, an appropriate no-pick-up time is essential to ensure construction efficiency and rapid drying and setting, while simultaneously maintaining adequate film quality. The self-developed anti-icing two-component road marking coating investigated in this study is a solvent-based system. Accordingly, the no-pick-up time was measured at 25°C.

#### Road performance test.

**Wear resistance test**

**Fig 5:**
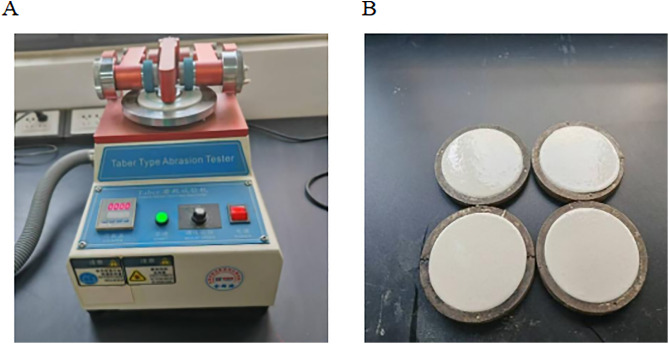
Wear resistance test diagram (A) Taber abrasion tester for wear resistance measurement; (B) schematic diagram of the coated test specimen.

The wear resistance of the coatings was evaluated in accordance with GB/T 1768–2006, Determination of Wear Resistance of Paints and Varnishes—Specification for the Rotating Rubber Grinding Wheel Method [18]. The test apparatus is shown in Fig 5. The Taber abrasion tester was purchased in Dongguan City, China, from Dongguan Huitai Instrument Equipment Co., Ltd. Circular steel plates with a diameter of 150 mm and a central hole were prepared, sequentially polished with sandpaper, rinsed with deionized water, and dried for 30 min. The marking coating was then applied to the prepared substrates, followed by the same curing procedure described previously.

After curing, the initial mass of each specimen was recorded. The coated plates were fixed on the abrasion tester turntable, and the test was conducted using rubber grinding wheels under a load of 500g per wheel at a rotation speed of 200r·min^-1^. Upon completion of the test, the abraded debris was removed, and the final mass of each specimen was measured. The wear loss rate was calculated according to formula 1:


$$L=\frac{m_{1}-m_{s}}{m_{1}}\times 100\%$$ (1)

In the formula:

*L* – Wear loss rate (g);

*m*_*1*_ – The original mass of the coating sample (g);

*m*_*s*_ – The mass of the coating sample after abrasion (g).

**Coefficient of retroreflection measure**

The most common method for incorporating glass beads into two-component road marking coatings is partial internal mixing. Internally mixed glass beads serve as a structural skeleton within the coating, while high-quality beads also contribute to surface protection. In China, road-marking glass beads are classified as No. 1, No. 2, and No. 3, with particle size increasing with bead number. According to GB/T 24722−2020, Road Marking Glass Beads [19], two-component, hot-melt, and water-based coatings are compatible with No. 1 glass beads. Accordingly, this study employed No. 1 glass beads supplied by Jiangsu Mingji New Materials Co., Ltd. (Table 3, Fig 5) to evaluate their key technical properties and retroreflective performance.

**Table 3:** No.1 glass beads. Physical and optical properties of glass beads used for retroreflection performance evaluation.

Test Item	Technical Requirements	Results/Remarks
Appearance	Shall be colorless, loose, spherical glass bodies.	Compliant
Sphericity	≥80%	≥92%
Particle Size	600–850 (μm)	20%
Density	2.4–4.3 (g/cm³)	2.8 (g/cm³)
Refractive Index	1.7 ≤ RI ≤ 1.9	1.81
Water Resistance	Glass beads shall not show fogging phenomenon in boiling water.	Compliant

**Fig 6:**
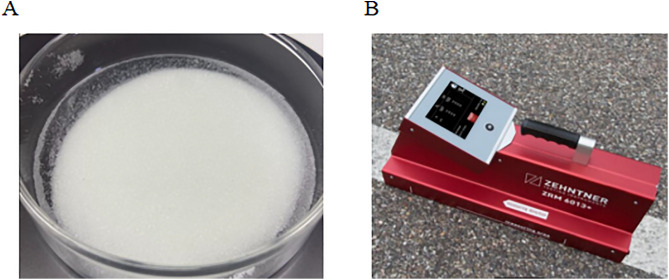
Test of retroreflection coefficient of marking coating. (A) Glass beads used for retroreflection; (B) handheld retroreflective coefficient tester.

The retroreflective performance of the marking coatings was evaluated in accordance with JT/T 690–2022, Test Method of Retroreflective Brightness Coefficient of Horizontal Coatings [20]. Coating specimens were applied to asbestos-free fiber cement plates, and glass beads were uniformly spread on the surface at a rate of 0.5 kg·cm^-2^. The retroreflective brightness coefficient (R_L_) of the markings was measured using a ZRM-6013 retroreflective coefficient tester (China, Shanghai, Shanghai Suio Instruments Co., Ltd.), as illustrated in Fig 6, for coatings containing different glass bead quantities.

**Coverage performance test**

**Fig 7:**
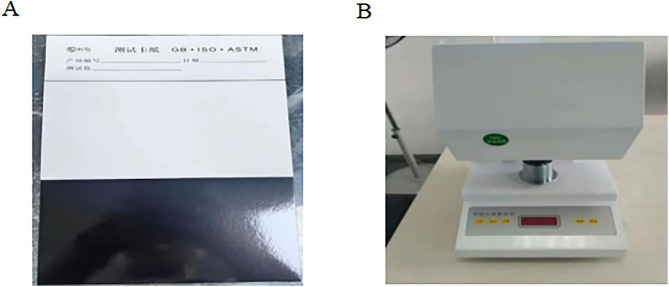
Coverage test paper and chromaticity tester. (A) Black-and-white contrast test paper; (B) chromaticity tester used for brightness measurement.


The coverage of the coatings was evaluated following the procedure specified in JT/T 280–2022, Pavement Marking Coating [21]. The test apparatus (China, Dongguan, Guangdong Nanjian Machinery Equipment Co., Ltd.) is shown in Fig 7. The self-developed two-component anti-icing road marking paint was thoroughly mixed and applied uniformly to black and white test substrates using a marking coater. After curing in a controlled environment for 24h, three measurement points were randomly selected on each side of the substrate. The brightness of the coating at these points was determined using a colorimeter, and the mean value of the three measurements was calculated. The average coverage was then determined according to the following formula 2:

$$H=\frac{B}{W}\times 100\%$$ (2)

In the formula:

*H* – Coating covering rate;

*B* – The average brightness of the marking paint on the black surface;

*W* – The average brightness of the marking paint on the white surface.

**Water resistance, alkaline performance test**

The water and alkali resistance of two-component road marking coatings are critical factors influencing the long-term durability of the markings. The anti-icing two-component coating samples were evaluated according to the water and alkali resistance test methods specified in JT/T 280–2022, Road Marking Coating [21].

**Fig 8:**
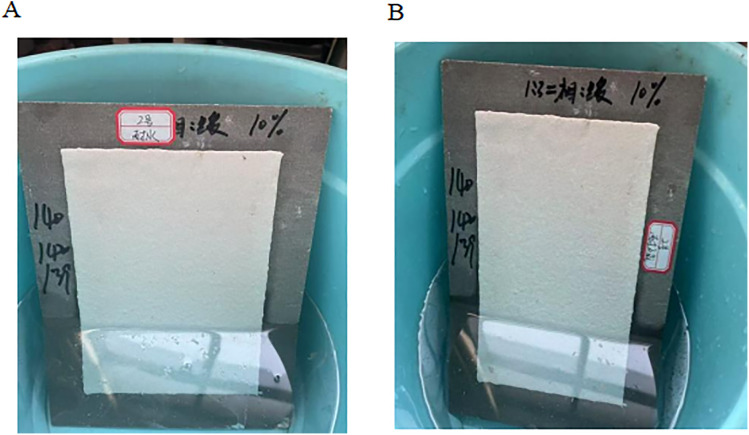
Water resistance, alkali resistance test. (A) Coating specimen after 24 h water immersion; (B) coating specimen after 24 h immersion in saturated Ca(OH)_2_ solution.

Water resistance: Using the immersion method, the coated test panels were placed in a plastic container filled to half capacity with water. After soaking for 24 h, the panels were inspected for signs of surface dulling, discoloration, blistering, wrinkling, or delamination. An example of the No. 2 marking paint panel after testing is shown in Fig 8(A).

Alkali resistance: Following the water resistance test, the coated panels were immersed in a saturated Ca(OH)_2_ solution for 24h. After exposure, the samples were examined for surface loss, discoloration, blistering, wrinkling, or delamination. An example of the No. 2 marking coating sample after testing is shown in Fig 8(B).

#### Microscopic analysis of ultraviolet aging.

**Anti-ultraviolet aging test**

Under natural conditions, the service life of road marking coatings is long and influenced by multiple environmental factors, making long-term aging tests challenging. Artificial accelerated aging using ultraviolet (UV) testing equipment can simulate the temperature, humidity, and UV radiation intensity experienced by road surfaces under natural conditions.

**Fig 9:**
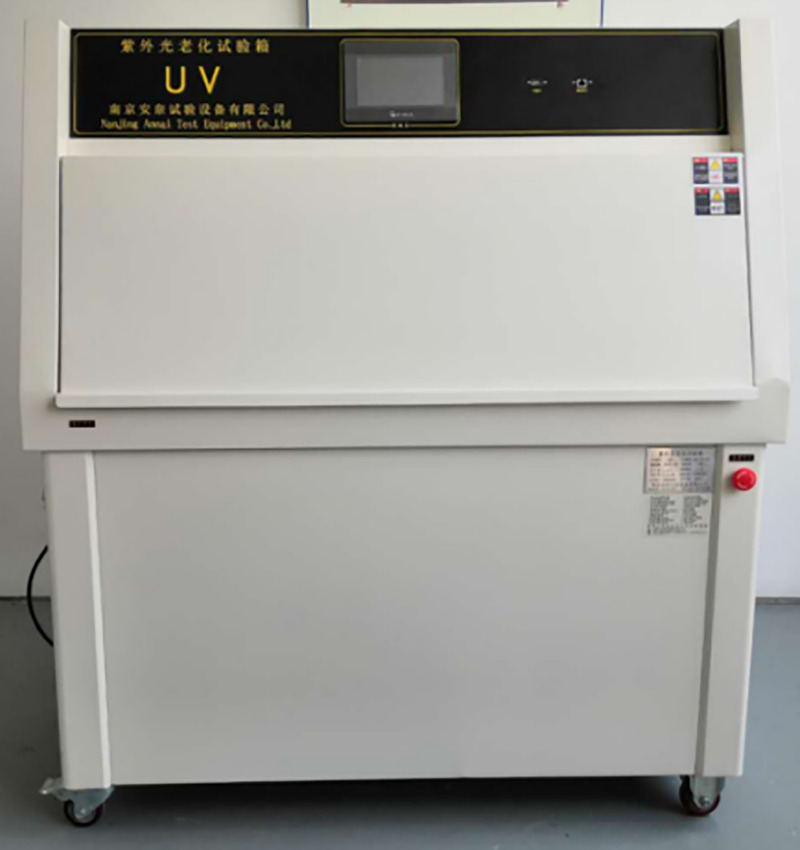
UV aging test chamber.

The UV aging performance of marking coatings No. 2−10 was evaluated following GB/T 14522−2008, Artificial Climate Aging Test Method for Plastics, Coatings, and Rubber Materials for Mechanical Industrial Products—Fluorescent Ultraviolet Lamp [22]. The accelerated aging cycle consisted of UVB-313 lamp irradiation at 60°C for 8h, followed by dark condensation at 50°C for 4 h. The total accelerated aging periods were 7, 14, and 21 days. Condensation on the samples was achieved by heating water to generate saturated water vapor, simulating wet conditions. The UV aging apparatus (China, Shenzhen, Xiya Instrumentation Equipment Co., Ltd.) is shown in Fig 9.

**Fig 10:**
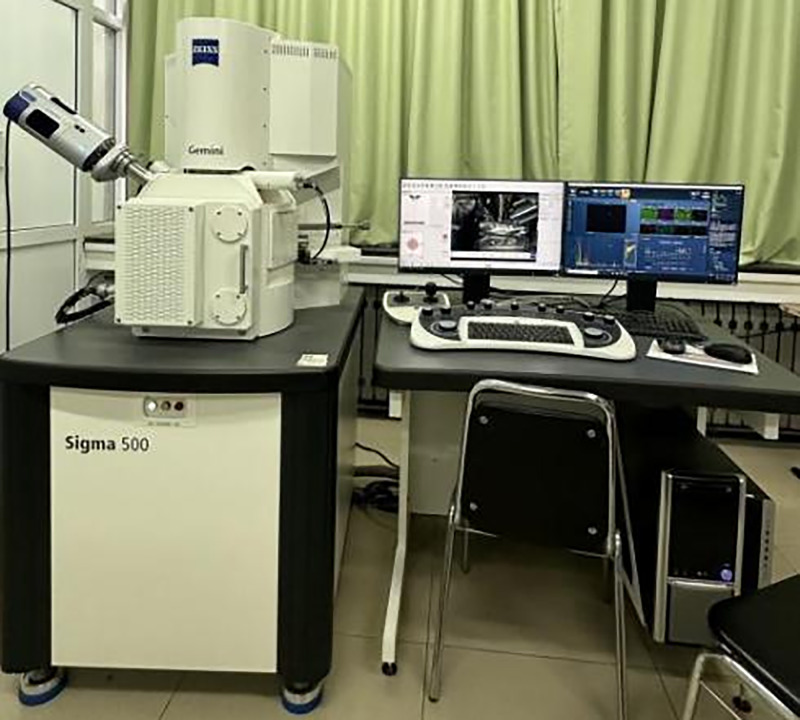
Scanning electron microscope.

The microstructure of the anti-icing two-component road marking coating samples before and after aging was examined using a SUPRA 55 SAPPHIRE scanning electron microscope (SEM; ZEISS, Germany), as shown in Fig 10. Since both PMMA resin and the coating are electrically non-conductive, the samples were mounted on conductive adhesive prior to imaging. Observations were conducted at a magnification of 500 times to assess the size and morphology of the coating.

#### Anti-icing and de-icing performance test.

**Ice adhesion pull-out test method**

**Fig 11:**
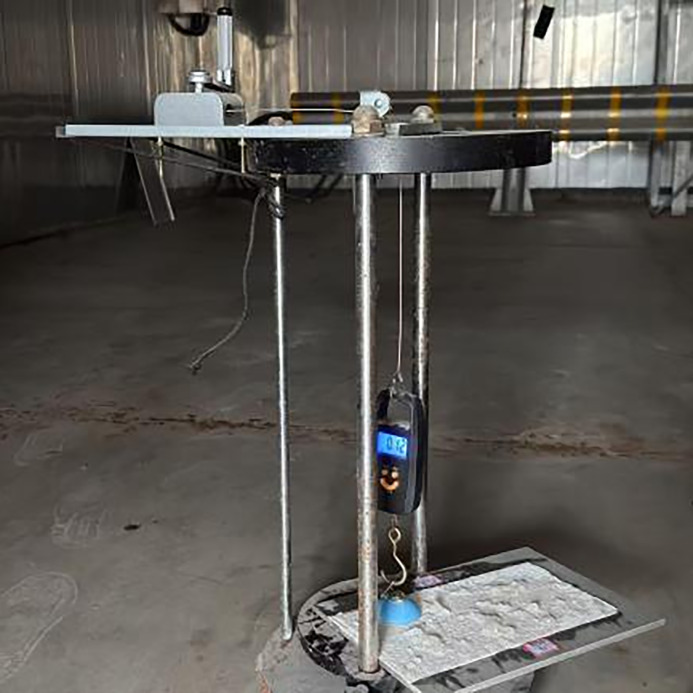
Ice adhesion pull-out test.

Ice adhesion strength tests were conducted in a walk-in climate simulation chamber to minimize the influence of ambient temperature. The testing setup and procedure are illustrated in Fig 11. The experimental steps were as follows:

① A pull-off stub was placed on the surface of the marking coating, and a waterproof gasket was installed around the outer ring of the stub to prevent water leakage.
② Using a syringe, 5 mL of water was injected into the stub through the reserved central hole.
③ The environmental chamber was set to −3°C, −6°C, and −9°C. The test panels were placed inside until the water fully froze.
④ After freezing, the panels were removed from the chamber. The hook of a manual pull-off tester was engaged with the top bend of the stub, and the handle was rotated to determine the ice adhesion strength between the ice layer and the marking coating.

**Freeze-thaw cycle protocol**

**Fig 12:**
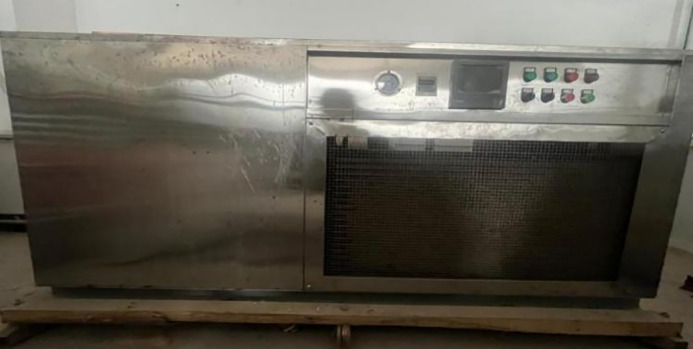
Freezing and thawing cycle test equipment.

The marked test panels were subjected to freeze-thaw cycling in a controlled environmental chamber, as shown in Fig 12. Each cycle consisted of a low -temperature phase at −20°C for 4h followed by a high-temperature phase at 20°C for 4h, resulting in an 8 h total cycle duration. Tests were conducted for 0, 10, and 20 cycles. After every 10 cycles, the panels were removed, and ice adhesion pull-off tests were performed to assess the bonding strength of the coating following the corresponding number of freeze-thaw cycles.

**Snow melting performance**

**Fig 13:**
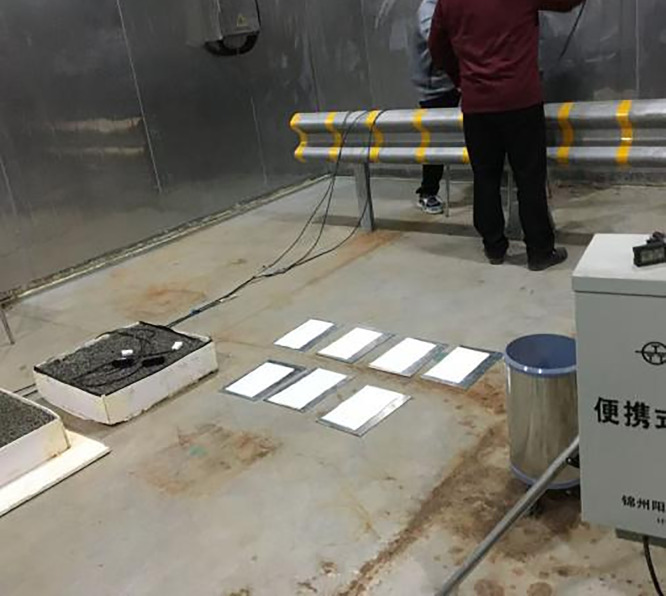
Snow melting performance test of marked lines.

The snow-melting performance of the anti-icing two-component road marking coating under winter low-temperature conditions was evaluated using a walk-in meteorological simulation chamber. The experimental procedure was as follows: the prepared coating test samples were placed inside the chamber, which was set to an ambient temperature range of −6–3°C with meteorological conditions simulating low temperature and snowfall. The chamber was then opened, and the snow-melting behavior on the surface of the samples was observed over time at different temperatures to assess the coating’s snow-melting performance, as shown in Fig 13.

### Measurement uncertainty

The uncertainties of the measurement instruments were considered to ensure the reliability of the experimental results. All instruments were calibrated according to the manufacturers’ specifications prior to testing. The measurement accuracy provided by the manufacturers was within the acceptable range specified in the relevant standards, and the experimental uncertainty did not affect the comparative analysis and observed performance trends.

All tests were conducted at least three times, and the reported values represent the average results. The experimental variability was within an acceptable range, indicating good repeatability.

## Results and discussion

### Effects of PCMs–salt addition on coating performance

**Fig 14:**
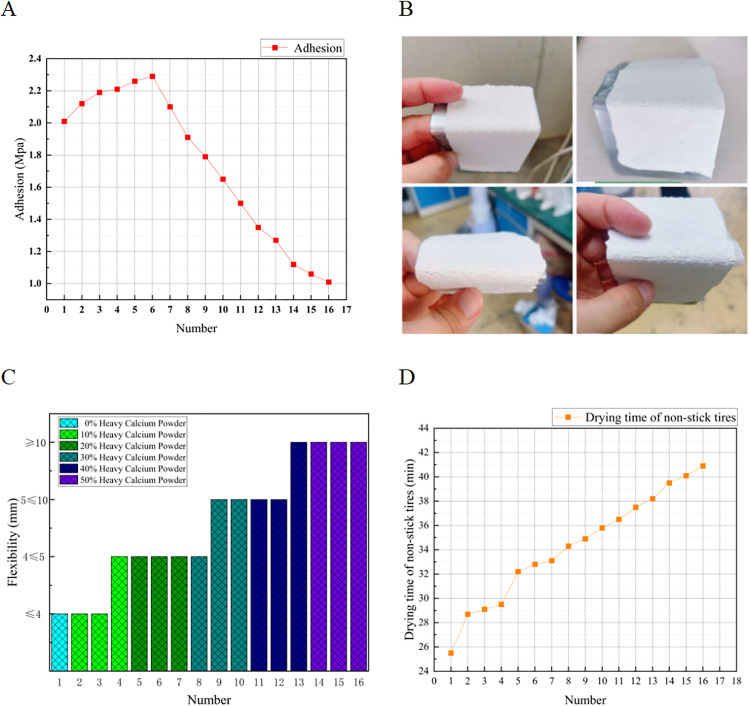
Test results of application performance test. The figure presents the results of the application performance tests, all of which were conducted using the 16 types of specimens listed in Table 2. **(A)** Adhesion strength of coatings with varying contents and TH-ME5/T-SEN ratios; (B) flexibility results expressed by bending diameter; (C) representative coating specimens after flexibility testing; and (D) non-stick tire time at 25°C. The results indicate that moderate incorporation of the PCM–salt composite (≤20 wt.%) improves or maintains adhesion and flexibility while keeping the drying time within specification limits, whereas excessive additive content leads to reduced adhesion, decreased flexibility, and prolonged non-stick tire time.

To investigate the influence of anti-icing materials on coating performance and to establish the relationship between these materials and the service performance of two-component road marking coatings, the prepared anti-icing coatings were evaluated for adhesion, flexibility, non-stick tire time, and other relevant properties. Based on the test data, the optimal formulation and proportions of the two-component coatings meeting the relevant specifications and standards were identified. The corresponding test results are presented in Fig 14.

As shown in Fig 14(A), the adhesion of the anti-icing two-component road marking coating initially increases and then decreases with increasing anti-icing material content. Within 0–20%, adhesion gradually improves, indicating good compatibility among the resin, pigments/fillers, and anti-icing material, as the resin can uniformly encapsulate and bind the components to form a stable coating structure. However, at 30–50% dosage, adhesion declines sharply; at 40–50%, it drops below 1.5 MPa, failing to meet the minimum requirement of JT/T 280–2022. This reduction is attributed to the low density of the TH-ME5 phase-change material: higher content increases the volume of pigments and fillers relative to the unchanged resin, causing particle agglomeration and reduced bonding strength.

Fig 14(B) shows that the bending radius increases with anti-icing material content, indicating reduced flexibility, with a pronounced step-change between 0–50%. At the same dosage, the bending radius is largest at a TH-ME5:T-SEN ratio of 3:1, demonstrating the significant influence of TH-ME5 on flexibility.

The non-stick tire drying time also increases with anti-icing content (Fig 14(D)). Within 10–20%, drying time rises moderately but remains within the JT/T 280–2022 limit of ≤35 min. At the same dosage, a TH-ME5:T-SEN ratio of 1:3 yields the shortest drying time, while higher TH-ME5 content prolongs it. Beyond 30% anti-icing material, drying time exceeds the specified value, indicating that content should be limited to ≤30%.

When the anti-icing material content is 0–30%, all performance indicators meet JT/T 280–2022 requirements. Above 30%, adhesion, flexibility, and non-stick tire time fail to comply, rendering further tests less meaningful. Optimal performance is achieved at a TH-ME5:T-SEN ratio of 3:1.

### Analysis on road performance

**Fig 15:**
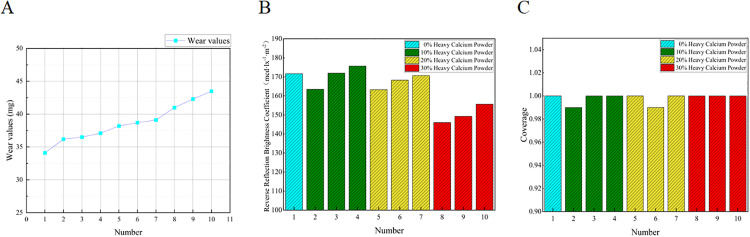
Test results of road performance. The figure presents the results of road performance tests, (A) Wear loss values of Specimens No. 1-10 measured by the rotating rubber grinding wheel method; (B) retroreflective brightness coefficient of markings containing internally mixed glass beads; and (C) coverage performance evaluated by brightness contrast between black and white substrates.

To further investigate the effect of anti-icing material content and ratio on the road performance of two-component road marking coatings, wear resistance, retroreflective coefficient, and coverage were selected as evaluation indicators. The self-developed anti-icing coatings were compared with standard two-component markings to assess how varying content and ratios influence performance. This study focused on coatings with 0–30% anti-icing material (Specimens No. 1–10). The corresponding test results are presented in Fig 15 and Table 4.

**Table 4:** Black coverage test results. Brightness values measured on black and white substrates for different coating formulations.

Number	Heavy Calcium Powder Replacement Ratio (%)	Phase Change Material Addition Ratio (%)	Slow-Release Salt Material Addition Ratio (%)	Luminance of Black Marking Paint (cd/m²)	Luminance of White Marking Paint (cd/m²)
1	0	0	0	80.16	80.03
2	10	25	75	80.43	80.46
3		50	50	80.30	80.20
4		75	25	80.30	80.16
5	20	25	75	80.23	80.23
6		50	50	80.10	80.23
7		75	25	80.16	80.06
8	30	25	75	80.26	80.26
9		50	50	80.20	80.20
10		75	25	80.13	80.10

As shown in Fig 15(A), the wear of the anti-icing two-component road marking coating increases with increasing substitution of heavy calcium powder. Within 10–20%, the increase is moderate, while above 20%, wear rises more sharply. At 30% dosage, the wear value remains below the maximum limit specified in JT/T 280–2022 (≤40 mg). The reduction in wear resistance at higher dosages is attributed to the effect of TH-ME5 and T-SEN on the cross-linking density of the resin-curing agent system. Within 10–20%, the wear remains within specification, indicating that the anti-icing material content should be controlled at ≤20% in subsequent tests.

Fig 15(B) shows that retroreflective brightness slightly increases with anti-icing material content. For the same dosage, a TH-ME5:T-SEN ratio of 3:1 yields slightly lower retroreflective performance than a 1:3 ratio, due to the lower density of TH-ME5, which reduces the embedding efficiency of glass beads and slightly weakens light reflection. At 10% content and a 3:1 ratio, the maximum retroreflective coefficient reaches 178 mcd·lx^-1^·m^-2^, 3% higher than the undoped coating. This improvement is attributed to the higher whiteness of the anti-icing material compared with heavy calcium powder, enhancing light scattering and retroreflection in combination with glass beads.

Coverage tests (Fig 15(C)) indicate minimal variation with increasing anti-icing content, demonstrating that the phase-change and salt-release materials are well-distributed and effectively fill gaps between pigment particles. The higher whiteness of the anti-icing material slightly improves the light absorption and scattering of the coating, maintaining good covering power.

### Water resistance, alkaline performance test

In actual service, road marking coatings are exposed to long-term water erosion, such as rainfall and immersion, and chemical attack from alkaline substrates, including cement and concrete. The water resistance test simulates prolonged water exposure to evaluate potential coating failures, such as blistering, peeling, discoloration, or dissolution. The alkali resistance test assesses the risk of chemical reactions between the coating and alkaline materials, including pulverization, cracking, and adhesion loss.

**Fig 16:**
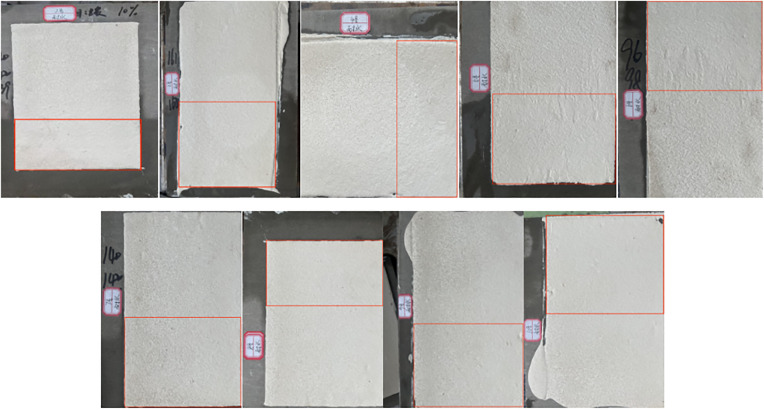
Sample of line 2-10 coating immersed in water for 24 hours. Surface appearance of marking coatings after 24h water immersion.

As shown in Fig 16 (red-framed immersion area), the surface of the marking coating before immersion exhibits a dense structure, with uniformly and stably dispersed filler particles. After 24 h of water immersion, no blistering, peeling, discoloration, or dissolution was observed, indicating effective cross-linking and curing. Water penetration into the coating is minimal, demonstrating that the self-developed two-component road marking coating possesses good water resistance.

**Fig 17:**
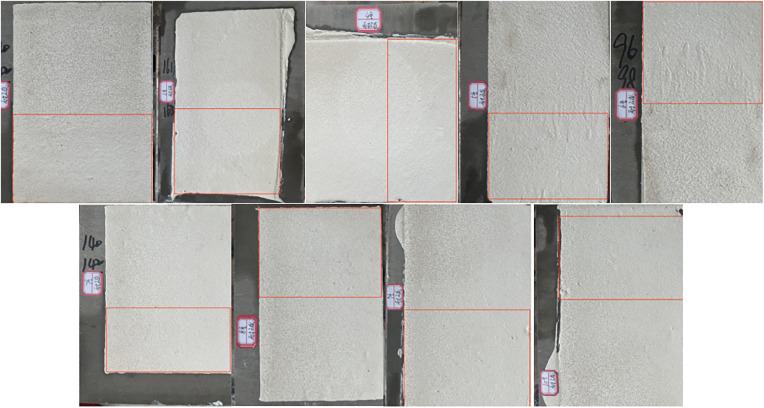
24 hour saturation of Ca(OH)_2_ in the sample of the 2–10 marking paint. Surface appearance of marking coatings after 24h immersion in saturated Ca(OH)_2_ solution.

Alkali resistance test for Specimens No. 2–10:

After 24 h immersion in a saturated Ca(OH)_2_ solution (red-framed area in Fig 17), the coating surface became slightly rough due to mild alkaline corrosion. No severe defects, such as visible holes or cracks, were observed. These results indicate that the self-developed anti-icing two-component road marking coating exhibits excellent alkali resistance, effectively resisting chemical corrosion and enhancing durability and service life under practical conditions.

### SEM test results

**Fig 18:**
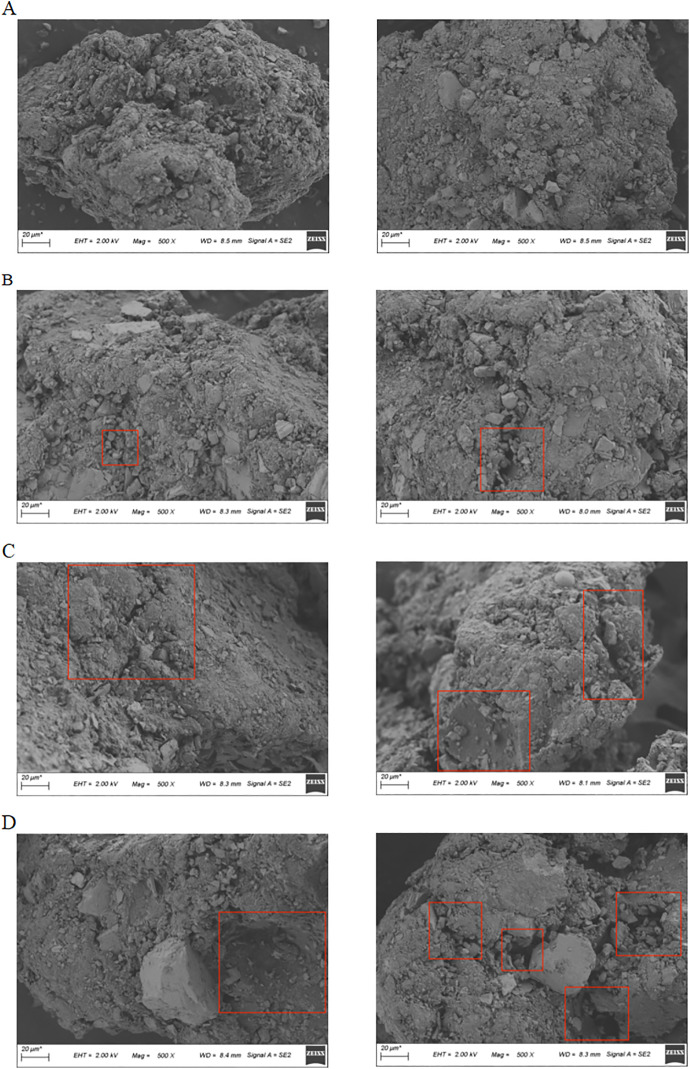
SEM images of anticoagulant ice two-component road marking coatings after different UV aging cycles. Scanning electron microscopy images of the selected coating (Specimen No. 2) after 0, 7, 14, and 21 days of UV aging, showing the microstructural evolution of the coating surface with increasing aging time.

Taking Specimen No. 2 as an example, the SEM images of the anti-icing two-component road marking coating before UV aging and after 0, 7, 14, and 21 days of accelerated aging are shown in Fig 18.

As shown in Fig 18(A), the SEM image before UV aging indicates that the anti-icing two-component road marking coating has a smooth and continuous surface, with uniformly distributed components and no signs of UV-induced damage. After 7 days of UV exposure (Fig 18(B)), the coating surface remains largely unchanged, suggesting that short-term UV radiation does not significantly affect its structure or physical properties.

With prolonged aging, however, Figs. 18(C) and 18(D) show that after 14 and 21 days, minor voids and fine cracks appear, and a few glass beads become detached. This is attributed to gradual resin degradation under UV radiation, leading to micro-crack formation, weakened bonding between pigment/filler particles and the matrix, and partial particle detachment. Despite these changes, the coating maintains overall integrity, demonstrating strong resistance to UV aging.

### Anti-icing and deicing performance

#### Analysis of ice adhesion pull-off test results.

The smooth surface of the road markings results in a relatively thin ice layer on the test panels, leading to low ice-coating adhesion. To illustrate the differences in ice adhesion between the anti-icing two-component coating and conventional coatings, as well as the effect of varying anti-icing material content and ratios, the pull-off test results are expressed as relative pull-off force. This parameter is defined as the ratio of the pull-off force of samples containing anti-icing materials to that of the unmodified reference sample, the calculation formula is given in formula 3:


$$I\;=\frac{L_{a}}{L_{b}}\times 100\%$$ (3)

In the formula:

*I* – Relative drawing force;

*L*_*a*_ - Drawing force of each dosage of marking paint template;

*L*_*b*_ - Drawing force of unmixed marking paint template.

**Fig 19:**
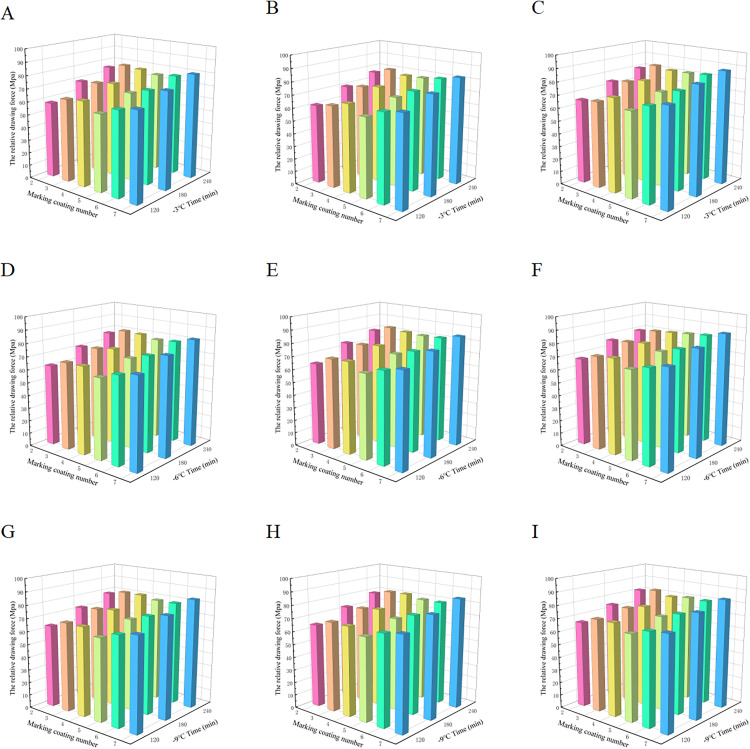
Relative Pulling Force Test Data Chart. Relative pull-off forces measured for Specimens No. 2-7 at −3°C, −6°C, and −9°C after 0, 10, and 20 freeze-thaw cycles, illustrating the effects of temperature and repeated freezing–thawing on ice adhesion behavior. (A) shows the results after 0 freeze–thaw cycles at −3°C, (B) shows the results after 10 freeze–thaw cycles at −3°C, (C) shows the results after 20 freeze–thaw cycles at −3°C, (D) shows the results after 0 freeze–thaw cycles at −6°C, (E) shows the results after 10 freeze–thaw cycles at −6°C, (F) shows the results after 20 freeze–thaw cycles at −6°C, (G) shows the results after 0 freeze–thaw cycles at −9°C, (H) shows the results after 10 freeze–thaw cycles at −9°C, (I) shows the results after 20 freeze–thaw cycles at −9°C.

It can be seen from Fig 19 that under constant temperature conditions, the relative drawing force of the specimen shows a significant upward trend with the extension of the standing time in the box, which is mainly due to the gradual attenuation of the latent heat energy storage effect of the phase change material over time. Taking the No.3 specimen at −3°C in Fig 19 as an example, when the standing time is extended from 120 min to 180 min, the relative drawing force of the specimen increases by 118.9%. When further extended to 240 min, the increase narrowed to 105%. This phenomenon shows that in the action period of the phase change material, the first 180 min is mainly the latent heat effect dominant stage, which significantly enhances the ice bond strength through the phase change heat absorption and release behavior; after more than 80 min, the phase change material is exhausted, and the anti-freezing performance is dominated by T-SEN type salts.

The results of the relative pull-out force test at the same ambient temperature show that the relative pull-out force of the specimen increases with the increase of the number of freeze-thaw cycles. This is mainly due to the gradual precipitation and depletion of salt ions in T-SEN during the freeze-thaw cycle, which eventually leads to a decrease in the anti-adhesion ability of the ice layer of the slow-release salt. When the content of anticoagulant ice material is fixed, the higher the proportion of T-SEN in the TH-ME5 and T-SEN blending system, the smaller the relative drawing force of the specimen. This is attributed to the fact that in the later stage of the action cycle, T-SEN undertakes the main anti-icing function, and the higher the content, the greater the consumption in the freeze-thaw cycle, which further verifies the attenuation law of T-SEN anti-adhesion efficiency.

From Fig 19 and the analysis above, it can be seen that under the same number of freeze-thaw cycles, when the content of anticoagulant ice material is 20% and the blending ratio of TH-ME5 and T-SEN is 1:3, the relative drawing force performance is better than other blending ratios, and the relative drawing force decreases with the decrease of temperature.

#### Snow melting performance results and analysis.

**Fig 20:**
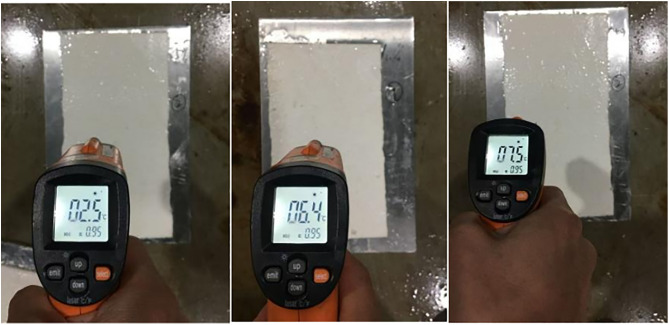
Surface temperature and snow melting of some marking paint samples. Observation of surface temperature variation and snow-melting performance at −3°C.

After the ambient temperature in the test chamber is reduced from 3°C to −3°C for a period of time, the snow melting on the surface of the specimen is observed in the test chamber, as shown in Fig 20.

It can be seen from Fig 20 above that when the ambient temperature in the test chamber drops to −3°C and the snow falls, it is observed that there is no snow on the surface of the marking coating, and the surface temperature of the marking coating is above zero. This is because the endothermic and exothermic phase change intervals of TH-ME5 phase change materials are −23.97°C ~ 5.26°C and −13.53°C ~ 6.52°C, respectively.When the ambient temperature reaches its endothermic and exothermic range, the latent heat performance of the phase change material will be excited at 0°C, and the cooling rate of the marking surface will be delayed, so that the marking coating has a good snow melting effect in this temperature range.

**Fig 21:**
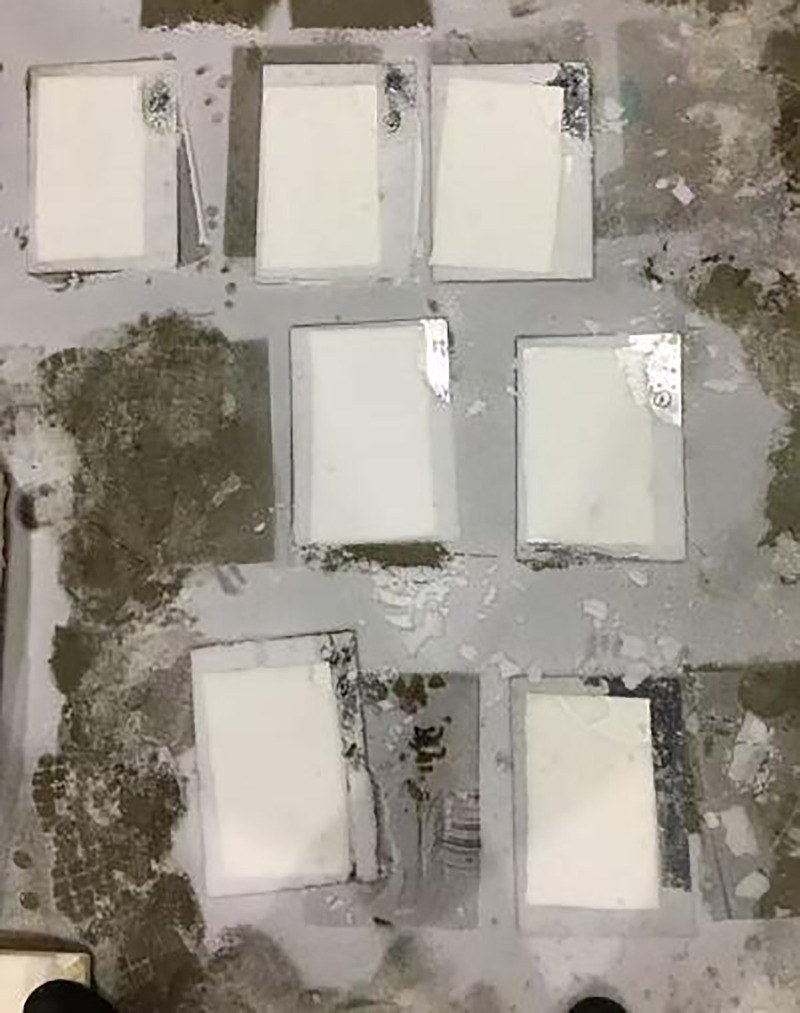
Snow melting on the surface of the −6 °C specimen. Comparison of snow accumulation and surface condition between marking coatings and the surrounding ground.

When the ambient temperature drops to −6°C and the ambient temperature lasts for a period of time, there is a small amount of snow on the surface of the ground and the marking coating in the observation test box. It can be seen from Fig 21 above that the surface of the marking coating has less snow than the ground, and the surface of the marking coating is softer after touching the snow. At the same time, it was observed that there was no ice layer on the surface of the marking paint, while the snow on the ground floor of the test chamber had a thin ice layer after the release of surface heat.

When the ambient temperature drops below −6°C, the salt analysis in T-SEN increases, and the precipitated salt ions are mixed with snow water to reduce the freezing point of snow water, slow down the condensation rate of the ice layer on the surface of the marking coating and reduce the hardness of the ice layer.

Therefore, in the early snow and light snow stage, the anti-condensation ice two-component road marking paint can melt a small amount of snow, slow down the appearance of ice on the surface of the marking, reduce the downward trend of the surface friction of the road marking under the low temperature condition in winter, and improve the safe traffic efficiency of vehicles in winter. To a certain extent, the hardness of the ice layer on the marking surface is reduced, the efficiency of anti-icing and snow removal by the subsequent road management department is improved, and the overall traffic efficiency of the road is improved.

The anti-icing performance observed in this study is consistent with recent reports on PCM-based and salt-based functional materials for cold-region infrastructure. Compared with conventional salt-releasing systems reported in recent studies, the proposed PCM-salt composite coating exhibits a more stable performance under repeated freeze-thaw cycles, indicating an improved durability. This validation confirms that the synergistic strategy adopted in this work provides a reliable enhancement rather than an isolated laboratory phenomenon.

## Discussion

The anti-icing and snow-melting performance of the proposed two-component road marking coating can be attributed to the synergistic interaction between phase-change materials (PCMs) and slow-release salts. The latent heat released during the phase transition of PCMs moderates surface temperature fluctuations and delays ice formation, while the gradual diffusion of salts lowers the freezing point of interfacial water and weakens ice-substrate adhesion.

Compared with single-mechanism approaches reported in recent studies, such as salt-only systems prone to rapid depletion or PCM-only systems limited by finite thermal capacity, the combined strategy adopted in this study provides a more stable and sustained anti-icing performance, particularly under repeated freeze–thaw conditions. This comparison confirms that the observed improvements are consistent with, and extend beyond, existing anti-icing concepts.

The durability results further validate the effectiveness of the proposed system. Many previously reported anti-icing coatings exhibit a pronounced loss of functionality after multiple freeze-thaw cycles due to salt leaching or microstructural degradation. In contrast, the PCM-salt modified coatings maintain a relatively stable anti-icing performance, indicating enhanced resistance to functional degradation. The controlled salt release mitigates rapid performance decay, while the PCM component alleviates thermal stress within the coating matrix, collectively contributing to improved long-term stability compared with conventional approaches.

In addition to functional effectiveness, the proposed coating maintains essential engineering properties required for practical road marking applications. The incorporation of PCMs and slow-release salts does not lead to unacceptable deterioration in wear resistance, retroreflectivity, or UV aging performance. Unlike many existing studies that primarily focus on deicing efficiency, this work provides an integrated validation framework that considers both anti-icing functionality and engineering applicability. The results demonstrate that the proposed PCM-salt composite system offers a balanced and scalable solution for durable anti-icing road markings in cold-region transportation infrastructure.

## Conclusion

This study systematically investigated the anti-icing and snow-melting performance of two-component road marking coatings incorporating phase-change materials (PCMs) and slow-release salts. Based on the experimental results, the following conclusions can be drawn:

- The incorporation of PCMs and slow-release salts significantly enhances the anti-icing performance of road marking coatings. The synergistic effect of latent heat release and gradual salt diffusion effectively reduces ice adhesion and delays ice formation under low-temperature conditions.
- Compared with conventional salt-based or PCM-only systems, the proposed composite coating exhibits improved durability. The anti-icing effectiveness can be maintained after repeated freeze-thaw cycles, indicating a more stable and long-lasting functional performance.
- Snow-melting experiments demonstrate that the combined PCM-salt system accelerates snow melting while maintaining acceptable mechanical properties, wear resistance, and retroreflectivity, which are essential for practical road marking applications.
- Microscopic observations and durability-related tests confirm that the addition of functional additives does not induce severe microstructural degradation, supporting the observed long-term performance stability of the coating system.

Several limitations of the present study should be acknowledged. The experimental evaluation was primarily conducted under laboratory-controlled conditions, which may not fully capture the complex environmental and traffic-related factors encountered in real road service scenarios. In addition, the long-term field performance, including salt release behavior over extended service periods and its potential environmental impact, was not addressed in this work.

Future research should therefore focus on large-scale field validation under actual climatic and traffic conditions to further assess the long-term anti-icing effectiveness and durability of the proposed system. Optimization of PCM content, salt release kinetics, and compatibility with different marking substrates should also be explored to balance performance, service life, and environmental sustainability.

This study provides a feasible and scalable strategy for developing durable anti-icing road marking materials, offering meaningful technical support for improving winter road safety in cold-region transportation infrastructure.

## Supporting information

S1 File To gain a detailed understanding of the experimental data collection and processing procedures, please refer to the file named *minimal data set* in the Raw Data folder of the supporting information. (DOCX)
