# Supplementary material for: Synergistic anti-icing and snow-melting performance of two-component road markings enabled by PCMs and slow-release salts
Source: PLoS One. 2026 Feb 2;21(2):e0341054. doi: 10.1371/journal.pone.0341054 (PMC12863552; doi:10.1371/journal.pone.0341054)
Supplement: S1 File — (DOCX) [file pone.0341054.s001.docx]

**minimal data set**

**Table 1 Types of anti-icing and snow melting two-component road marking specimens（Table 2 in the paper）**

| **Number** | **Heavy Calcium Powder Replacement Ratio (%)** | **Phase Change Material Addition Ratio (%)** | **Slow-Release Salt Material Addition Ratio (%)** |
| --- | --- | --- | --- |
| 1 | 0 | 0 | 0 |
| 2 | 10 | 25 | 75 |
| 3 |  | 50 | 50 |
| 4 |  | 75 | 25 |
| 5 | 20 | 25 | 75 |
| 6 |  | 50 | 50 |
| 7 |  | 75 | 25 |
| 8 | 30 | 25 | 75 |
| 9 |  | 50 | 50 |
| 10 |  | 75 | 25 |
| 11 | 40 | 25 | 75 |
| 12 |  | 50 | 50 |
| 13 |  | 75 | 25 |
| 14 | 50 | 25 | 75 |
| 15 |  | 50 | 50 |
| 16 |  | 75 | 25 |

Table 2 Data Sheet for Adhesion Test of Marking Coating （Figure 14(a) in the paper）

| Number | Heavy Calcium Powder Replacement Ratio (%) | Phase Change Material Addition Ratio (%) | Slow-Release Salt Material Addition Ratio (%) | Adhesion diagram  （Mpa） |
| --- | --- | --- | --- | --- |
| 1 | 0 | 0 | 0 | 2.01 |
| 2 | 10 | 25 | 75 | 2.12 |
| 3 |  | 50 | 50 | 2.19 |
| 4 |  | 75 | 25 | 2.21 |
| 5 | 20 | 25 | 75 | 2.26 |
| 6 |  | 50 | 50 | 2.29 |
| 7 |  | 75 | 25 | 2.10 |
| 8 | 30 | 25 | 75 | 1.91 |
| 9 |  | 50 | 50 | 1.79 |
| 10 |  | 75 | 25 | 1.65 |
| 11 | 40 | 25 | 75 | 1.50 |
| 12 |  | 50 | 50 | 1.35 |
| 13 |  | 75 | 25 | 1.27 |
| 14 | 50 | 25 | 75 | 1.12 |
| 15 |  | 50 | 50 | 1.06 |
| 16 |  | 75 | 25 | 1.01 |

Table 3 Flexibility Test of Marking Coating（Figure 14(c) in the paper）

| Number | Heavy Calcium Powder Replacement Ratio (%) | Phase Change Material Addition Ratio (%) | Slow-Release Salt Material Addition Ratio (%) | Flexibility（mm） |
| --- | --- | --- | --- | --- |
| 1 | 0 | 0 | 0 | ≦4 |
| 2 | 10 | 25 | 75 | ≦4 |
| 3 |  | 50 | 50 | ≦4 |
| 4 |  | 75 | 25 | 4≦5 |
| 5 | 20 | 25 | 75 | 4≦5 |
| 6 |  | 50 | 50 | 4≦5 |
| 7 |  | 75 | 25 | 4≦5 |
| 8 | 30 | 25 | 75 | 4≦5 |
| 9 |  | 50 | 50 | 5≦10 |
| 10 |  | 75 | 25 | 5≦10 |
| 11 | 40 | 25 | 75 | 5≦10 |
| 12 |  | 50 | 50 | 5≦10 |
| 13 |  | 75 | 25 | ≧10 |
| 14 | 50 | 25 | 75 | ≧10 |
| 15 |  | 50 | 50 | ≧10 |
| 16 |  | 75 | 25 | ≧10 |

Table 4 Drying time of non stick tire sample at 25 ℃（Figure 14(d) in the paper）

| Number | Heavy Calcium Powder Replacement Ratio (%) | Phase Change Material Addition Ratio (%) | Slow-Release Salt Material Addition Ratio (%) | Drying time  （min） |
| --- | --- | --- | --- | --- |
| 1 | 0 | 0 | 0 | 25.5 |
| 2 | 10 | 25 | 75 | 28.7 |
| 3 |  | 50 | 50 | 29.1 |
| 4 |  | 75 | 25 | 29.5 |
| 5 | 20 | 25 | 75 | 32.2 |
| 6 |  | 50 | 50 | 32.8 |
| 7 |  | 75 | 25 | 33.1 |
| 8 | 30 | 25 | 75 | 34.3 |
| 9 |  | 50 | 50 | 34.9 |
| 10 |  | 75 | 25 | 35.8 |
| 11 | 40 | 25 | 75 | 36.5 |
| 12 |  | 50 | 50 | 37.5 |
| 13 |  | 75 | 25 | 38.2 |
| 14 | 50 | 25 | 75 | 39.5 |
| 15 |  | 50 | 50 | 40.1 |
| 16 |  | 75 | 25 | 40.9 |

Table 5 Wear values of test samples（Figure 15(a) in the paper）

| Number | Heavy Calcium Powder Replacement Ratio (%) | Phase Change Material Addition Ratio (%) | Slow-Release Salt Material Addition Ratio (%) | Wear values  （mg） |
| --- | --- | --- | --- | --- |
| 1 | 0 | 0 | 0 | 34.1 |
| 2 | 10 | 25 | 75 | 36.2 |
| 3 |  | 50 | 50 | 36.5 |
| 4 |  | 75 | 25 | 37.1 |
| 5 | 20 | 25 | 75 | 38.2 |
| 6 |  | 50 | 50 | 38.7 |
| 7 |  | 75 | 25 | 39.1 |
| 8 | 30 | 25 | 75 | 41.0 |
| 9 |  | 50 | 50 | 42.3 |
| 10 |  | 75 | 25 | 43.5 |

Table 6 Results of Reverse Reflection Brightness Coefficient Test（Figure 15(b) in the paper）

| Number | Heavy Calcium Powder Replacement Ratio (%) | Phase Change Material Addition Ratio (%) | Slow-Release Salt Material Addition Ratio (%) | Retroreflection coefficient  （mcd·lx^-1^·m^-2^） | | | Mean value of inverse reflectance luminance coefficient（mcd·lx^-1^·m^-2^） |
| --- | --- | --- | --- | --- | --- | --- | --- |
| 1 | 0 | 0 | 0 | 171 | 172 | 172 | 171.7 |
| 2 | 10 | 25 | 75 | 175 | 176 | 176 | 163.5 |
| 3 |  | 50 | 50 | 172 | 172 | 172 | 172.0 |
| 4 |  | 75 | 25 | 165 | 166 | 165 | 175.7 |
| 5 | 20 | 25 | 75 | 170 | 171 | 171 | 163.3 |
| 6 |  | 50 | 50 | 168 | 168 | 169 | 168.3 |
| 7 |  | 75 | 25 | 163 | 162 | 165 | 170.7 |
| 8 | 30 | 25 | 75 | 154 | 157 | 156 | 146.0 |
| 9 |  | 50 | 50 | 149 | 150 | 149 | 149.3 |
| 10 |  | 75 | 25 | 146 | 146 | 146 | 155.7 |

Table 7 Coverage Calculation Results （Figure 15(c) in the paper）

| Number | Heavy Calcium Powder Replacement Ratio (%) | Phase Change Material Addition Ratio (%) | Slow-Release Salt Material Addition Ratio (%) | Coating coverage rate |
| --- | --- | --- | --- | --- |
| 1 | 0 | 0 | 0 | 1.00 |
| 2 | 10 | 25 | 75 | 0.99 |
| 3 |  | 50 | 50 | 1.00 |
| 4 |  | 75 | 25 | 1.00 |
| 5 | 20 | 25 | 75 | 1.00 |
| 6 |  | 50 | 50 | 0.99 |
| 7 |  | 75 | 25 | 1.00 |
| 8 | 30 | 25 | 75 | 1.00 |
| 9 |  | 50 | 50 | 1.00 |
| 10 |  | 75 | 25 | 1.00 |

Table 8 -3 ℃ Relative Pulling Force Test Results（Figure 19 in the paper）

| No freeze-thaw cycle | | | | | 10 freeze-thaw cycles | | | | | | 20 freeze-thaw cycles | | | | | |  |
| --- | --- | --- | --- | --- | --- | --- | --- | --- | --- | --- | --- | --- | --- | --- | --- | --- | --- |
| 120min | 180min | 240min | | 120min | | | 180min | | 240min | | 120min | 180min | | 240min | |  |  |
| 58.2 | 69.2 | | 75.5 | | | 61.2 | | 70.1 | | 76.7 | 65.3 | | 74.2 | | 80.1 | | |
| 63.8 | 70.4 | | 79.2 | | | 63.8 | | 72.4 | | 80.9 | 66.9 | | 76.4 | | 84.2 | | |
| 65.6 | 72.5 | | 78.2 | | | 68.2 | | 74.7 | | 78.2 | 72.6 | | 79.5 | | 82.3 | | |
| 59.1 | 67.9 | | 76.1 | | | 61.5 | | 69.3 | | 78.6 | 65.9 | | 73.4 | | 82.5 | | |
| 65.2 | 72.5 | | 77.6 | | | 68.1 | | 76.5 | | 80.3 | 72.3 | | 76.7 | | 83.4 | | |
| 68.2 | 74.9 | | 81.2 | | | 70.5 | | 77.1 | | 83.4 | 75.6 | | 84.0 | | 88.5 | | |

Table 9 -6 ℃ Relative Pulling Force Test Results（Figure 19 in the paper）

| No freeze-thaw cycle | | | | | 10 freeze-thaw cycles | | | | | | 20 freeze-thaw cycles | | | | | |  |
| --- | --- | --- | --- | --- | --- | --- | --- | --- | --- | --- | --- | --- | --- | --- | --- | --- | --- |
| 120min | 180min | 240min | | 120min | | | 180min | | 240min | | 120min | 180min | | 240min | |  |  |
| 62.3 | 71.3 | | 77.5 | | | 63.6 | | 74.2 | | 79.5 | 67.5 | | 76.5 | | 79.5 | | |
| 67.4 | 72.41 | | 81.2 | | | 70.1 | | 75.2 | | 83.9 | 72.1 | | 77.6 | | 81.1 | | |
| 67.6 | 74.5 | | 80.6 | | | 70.8 | | 76.8 | | 82.3 | 73.5 | | 78.9 | | 82.1 | | |
| 62.1 | 69.9 | | 78.5 | | | 65.1 | | 73 | | 81.6 | 68.1 | | 75 | | 83.2 | | |
| 67.2 | 74.5 | | 79.3 | | | 70.2 | | 77.5 | | 82.1 | 72.2 | | 79.3 | | 84.2 | | |
| 70.1 | 77.2 | | 83.1 | | | 73.4 | | 80.2 | | 85.4 | 75.6 | | 82.3 | | 87.6 | | |

Table 10 -9 ℃ Relative Pulling Force Test Results（Figure 19 in the paper）

| No freeze-thaw cycle | | | | | | 10 freeze-thaw cycles | | | | | | 20 freeze-thaw cycles | | | | | |
| --- | --- | --- | --- | --- | --- | --- | --- | --- | --- | --- | --- | --- | --- | --- | --- | --- | --- |
| 120min | 180min | | 240min | | 120min | | 180min | | 240min | | 120min | | 180min | | 240min | |  |
| 63.5 | | 72.1 | | 78.6 | | 64.3 | | 72.5 | | 78.9 | | 66.2 | | 74.3 | | 81.2 | |
| 68.6 | | 73.4 | | 81.5 | | 69.1 | | 73.8 | | 81.9 | | 71.3 | | 74.2 | | 83.1 | |
| 68.5 | | 75.1 | | 81.5 | | 68.9 | | 75.6 | | 82.2 | | 71.5 | | 77.6 | | 80.1 | |
| 63.4 | | 70.5 | | 79.4 | | 64.1 | | 71.0 | | 80.0 | | 66.2 | | 72.6 | | 81.5 | |
| 68.5 | | 75.4 | | 79.5 | | 69.6 | | 76.1 | | 80.1 | | 70.9 | | 77.0 | | 81.2 | |
| 71.3 | | 78.4 | | 84.5 | | 71.8 | | 79.1 | | 85.1 | | 72.3 | | 80.5 | | 84.5 | |
